# Risk factors for periprosthetic joint infection following primary total knee arthroplasty: a systematic review and meta-analysis

**DOI:** 10.3389/fsurg.2025.1715571

**Published:** 2026-01-08

**Authors:** Jiashun Li, Zhen Liu, Zheng Wang, Deyu Li, Heng Zhang, Shuo Hao, Qingxue Zhu, Maihemuti Yakufu, Ainikaer Abulaiti, Abuduwupuer Haibier

**Affiliations:** 1 Sixth Affiliated Hospital of Xinjiang Medical University, Orthopedic Hospital of Xinjiang Uygur Autonomous Region, Urumqi, China; 2 XinJiang Medical University, Urumqi, Xinjiang, China; 3 Fifth Affiliated Hospital of Xinjiang Medical University, Orthopedic Hospital of Xinjiang Uygur Autonomous Region, Urumqi, China; 4 People’s Hospital of Xinjiang Uygur Autonomous Region, Urumqi, China

**Keywords:** meta-analysis, periprosthetic joint infection, PJI, risk factors, TKA, total knee arthroplasty

## Abstract

**Objective:**

Periprosthetic joint infection (PJI) represents a severe and devastating complication following primary total knee arthroplasty (TKA), leading to higher morbidity and implant failure. However, existing evidence regarding its risk factors remains inconsistent.

**Method:**

PubMed, Cochrane Library, Embase, China National Knowledge Infrastructure (CNKI), Wanfang Database, VIP Chinese Journal Database, and the Chinese Biomedical Literature Service System (SinoMed) were searched for primary studies from the database inception to August 2025. Data were managed with EndNote X9 software, meta-analyses were performed using Review Manager 5.4, infection risk was analyzed using STATA 18, and the comprehensive incidence rate of PJI was analyzed using R 4.5.1.

**Results:**

A total of 1,569 studies were identified, of which 20 were included in this systematic review. The meta-analysis including 20 studies analyzed eight risk factors: longer operation time (OR = 9.10, 95% CI: 7.66–10.80), obesity (OR = 13.95, 95% CI: 12.06–16.14), male gender (OR = 2.67, 95% CI: 1.80–3.95), diabetes (OR = 2.98, 95% CI: 2.27–3.92), longer hospital stay (OR = 1.73, 95% CI: 1.38–2.16), use of immunosuppressants (OR = 5.76, 95% CI: 2.77–11.98), hypoalbuminemia (OR = 6.24, 95% CI: 4.00–9.73), and underlying systemic inflammatory disease (OR = 3.47, 95% CI: 1.92–6.27). All identified risk factors were associated with an increased risk of developing PJI after primary TKA.

**Conclusion:**

The meta-analysis confirmed that longer operative time, obesity, male gender, diabetes, longer hospital stay, use of immunosuppressants, hypoalbuminemia, and underlying systemic inflammatory disease are risk factors for PJI after primary TKA. This systematic review and meta-analysis provide Level II evidence (according to the Oxford Centre for Evidence-Based Medicine criteria) for the identified risk factors.

## 1 Introduction

Total knee arthroplasty (TKA) is a highly successful and cost-effective surgical intervention for end-stage knee osteoarthritis, significantly alleviating pain and restoring function in millions of patients worldwide (1, 2). With the aging global population and rising prevalence of osteoarthritis, the annual volume of TKA procedures continues to increase. However, this success is tempered by the risk of postoperative complications, among which periprosthetic joint infection (PJI) remains one of the most devastating and challenging. PJI is the leading cause of early revision TKA, accounting for approximately 25%–30% of all revision cases (3–6), and it poses a tremendous burden on healthcare systems, with treatment costs often exceeding those of the primary procedure by severalfold (7).

The reported incidence of PJI following primary TKA varies in the literature but is generally estimated to be between 1% and 2% (8–10). Although this percentage may seem modest, it corresponds to a substantial absolute number of affected patients due to the high procedural volume, making PJI a significant public health concern. The consequences for affected patients are severe and can include prolonged antibiotic therapies, multiple complex surgical procedures (including debridement, antibiotics, and implant retention, one- or two-stage revision), extended rehabilitation, and often a permanent functional deficit (11–13). Furthermore, PJI is associated with a significantly higher mortality rate compared with aseptic revision procedures (14).

Given the profound implications of PJI, identifying at-risk patients is paramount for implementing targeted preemptive strategies. Numerous studies have investigated potential risk factors, which can be broadly categorized as patient-specific modifiable (e.g., obesity, diabetes mellitus, and malnutrition) and non-modifiable factors (e.g., age, gender, and rheumatoid arthritis), as well as procedure-related and healthcare system-related factors. However, evidence from individual studies is often inconsistent or underpowered. Therefore, we conducted a systematic review and meta-analysis to synthesize the evidence and establish the risk factors for developing PJI after primary TKA.

## 2 Method

### 2.1 Study search strategy

The PubMed, Cochrane Library, Embase, China National Knowledge Infrastructure (CNKI), Wanfang Database, VIP Chinese Journal Database, and the Chinese Biomedical Literature Service System (SinoMed) from their inception to August 2025. A combined search strategy using both subject headings and free-text terms was employed. The search keywords included: total knee arthroplasty, TKA, prosthesis, periprosthetic infection, PJI, and risk factors. Gray literature (conference abstracts, doctoral theses, master's theses) obtained from the Chinese databases CNKI, Wanfang, and VIP was also considered. The complete search strategy is provided in Supplementary Text S1.

### 2.2 Diagnostic criteria for PJI

According to the International Musculoskeletal Infection Society (MSIS), the International Consensus Meeting (ICM), and the European Bone and Joint Infection Society (EBJIS), PJI is defined as follows:

Major criteria: Presence of a sinus tract communicating with the prosthesis, or isolation of the same pathogen from two or more separate samples of synovial fluid or intraoperative tissue cultures. Minor criteria: Serological markers [significantly elevated c-reactive protein (CRP) and erythrocyte sedimentation rate (ESR)], synovial fluid analysis [white blood cell count >3,000/µL or polymorphonuclear neutrophil (PMN) percentage >80%], intraoperative findings (purulent secretion or suppuration around the prosthetic tissue), histopathological examination (>5 polymorphonuclear leukocytes per high-power field), and emerging biomarkers (positive synovial α-defensin or D-dimer), meeting any single major criterion confirms the diagnosis (3).

### 2.3 Inclusion and exclusion criteria

Four authors (JL, ZL, ZW, DL) independently determined study eligibility. Any difference in opinion regarding eligibility was resolved through consensus.

Inclusion criteria: (Ⅰ) Study subjects were adult patients who underwent primary total knee arthroplasty at the same hospital. (Ⅱ) The exposure factor is one or more risk factors potentially associated with PJI after primary TKA, such as obesity and diabetes. (Ⅲ) The control group consists of patients who are not exposed to the risk factors under study. (Ⅳ) The outcome was the occurrence of periprosthetic joint infection after primary total knee arthroplasty. (Ⅴ) Patients were followed up for at least 6 months. (Ⅵ) The study design is a cohort study or case–control study, with the research focus on validating the risk of PJI infection following primary TKA.

Exclusion criteria: (Ⅰ) Patients not hospitalized during the whole study period; (Ⅱ) patients undergoing revision total knee arthroplasty; (Ⅲ) studies with missing medical data; (Ⅳ) duplicate publications, letters, or conference papers; (Ⅴ) animal studies, review articles, and systematic reviews; and (Ⅵ) studies not related to total knee arthroplasty.

### 2.4 Study selection

All the studies containing abstracts and titles were imported to EndNote X9. After removing duplicate papers, three investigators (JL, ZL, ZW) independently screened titles and abstracts according to the inclusion and exclusion criteria and then further screened the initially included literature by reading the full text to exclude those that do not meet the inclusion criteria. In cases of any disagreement in this step, a fourth investigator (DL) performed the same screening process as described above, and a consensus was reached through discussion.

### 2.5 Data abstraction and validity assessment

For studies meeting the inclusion criteria, the following information was extracted: first author, publication year, study design type, sample size, evaluated variables or risk factors, odds ratio, and 95% CI.

### 2.6 Quality assessment of studies

The methodological quality of the included literature was evaluated using the Newcastle–Ottawa scale (NOS) to assess the quality of all included studies.

### 2.7 Inclusion in the meta-analysis, data extraction, and statistical methods

Meta-analysis on the included studies was performed using Review Manager 5.4. The mean values and their standard deviations were extracted from each study to calculate the weighted mean difference and 95% confidence interval (CI), as well as the pooled odds ratio (OR) and 95% CI. For the heterogeneity test model selection, the Cochrane's *Q* test was employed to evaluate the degree of heterogeneity among studies. When heterogeneity was low (e.g., *I*^2^ < 50%) or *P*-value >0.10, indicating that the differences were mainly due to sampling error, a fixed-effects model was used. When heterogeneity was high (e.g., *I*^2^ ≥ 50%) or *P*-value ≤0.10, indicating the presence of genuine differences among studies, a random-effect model was used. The pooled odds ratios (ORs) and 95% confidence intervals (CIs) were calculated for each risk factor for PJI following primary TKA, including longer operation time, obesity, male gender, diabetes, longer hospital stay, use of immunosuppressants, hypoalbuminemia, and underlying systemic inflammatory disease. A significance level of *P* < 0.05 was adopted to indicate statistical significance. Meta-analysis was performed using both fixed-effects and random-effects models.

## 3 Results

### 3.1 Search results

**Figure 1:**
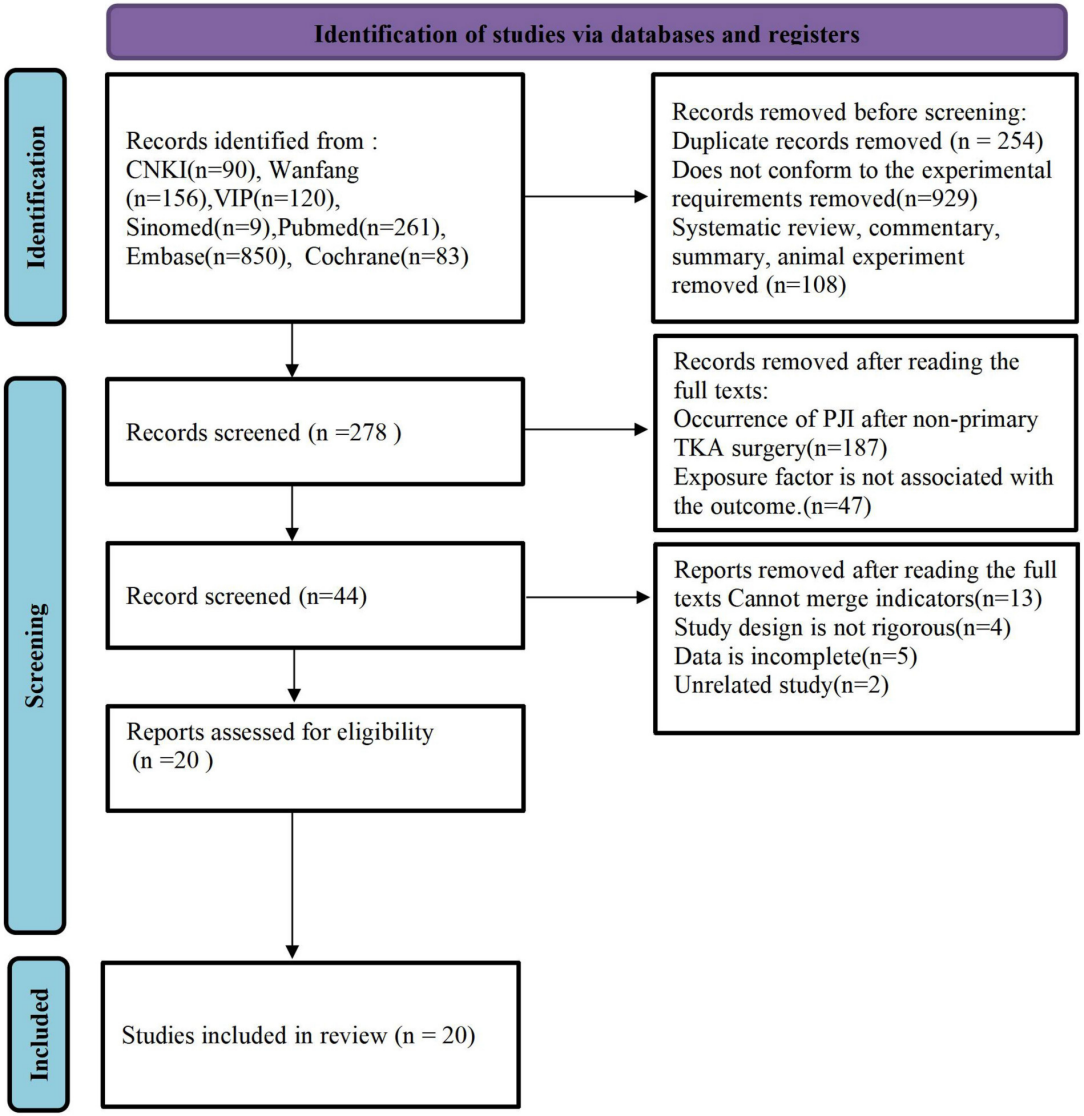
Flow diagram of study selection (Supplementary Text S2).

Figure 1 shows the study selection process. After excluding 254 duplicate studies, 1,037 were then excluded based on abstract and title screening, and an additional 278 studies were discarded after full-text review. Therefore, 20 studies reporting PJI following primary TKA were included.

### 3.2 Characteristics of the studies included

Table 1 provides a detailed description of the studies analyzed and lists the risk factors identified in each study. The 20 studies included were published from database inception to August 2025. Among them, 13 were cohort studies (15–17, 19–27, 34), and 7 were case–control studies (18, 28–33). Three studies were conducted in the USA (15, 23, 25), 2 in Australia (16, 17), 1 in Pakistan (19), 1 in Finland (20), 1 in Germany (21), 1 in Japan (24), and 11 in China (18, 22, 26–34).

**Table 1:** Characteristics of the studies included.

Study inclusion	Publication year	Country	Study type	Number of cases	Total sample size	Incidence of PJI (%)	Risk factors
Anis et al. (15)	2019	USA	Cohort	79	11,840	0.7	1, 2
Armit et al. (16)	2018	Australia	Cohort	29	1,058	2.7	3, 4, 5, 6
Dowsey and Choong (17)	2009	Australia	Cohort	18	1,214	1.5	2, 3, 7
Guo et al. (18)	2020	China	CC	54	162	33.33	1, 2, 14, 15, 16, 17, 18
Iqbal et al. (19)	2020	Pakistan	Cohort	48	4,269	1.1	1, 2, 19
Jämsen et al. (20)	2012	Finland	Cohort	31	3,915	0.79	2, 7
Claus et al. (21)	2006	German	Cohort	144	17,641	0.8	3, 6, 27, 28
Siu et al. (22)	2018	China	Cohort	34	2,543	1.34	20
Remily et al. (23)	2023	USA	Cohort	32,093	13,80,536	2.32	2, 7, 29, 30, 31, 32
Suzuki et al. (24)	2011	Japan	Cohort	17	2,022	0.84	2, 3, 15
Wang et al. (25)	2019	USA	Cohort	70	7,861	0.89	1, 2, 31, 34
Cheng et al. (26)	2022	China	Cohort	14	525	2.67	7, 10, 27
Ning et al. (27)	2017	China	Cohort	12	1,249	0.96	1, 2, 7, 13, 17, 20
Baoyu (28)	2016	China	CC	15	45	33.33	1, 2, 13, 14, 15, 16, 17, 18, 21
Di (29)	2020	China	CC	14	42	33.33	1, 2, 7, 14, 16, 17
Tao et al. (30)	2017	China	CC	22	402	5.47	3, 10, 24, 25
Zhe et al. (31)	2021	China	CC	21	105	20	1, 16, 22, 23
Shaoqiang et al. (32)	2024	China	CC	69	276	25	1, 7, 17, 20, 26
Zhongren (33)	2023	China	CC	46	131	35.11	7, 22, 24
Du (34)	2022	China	Cohort	36	868	4.15	2, 17, 24, 36, 37

1, longer operative time; 2, obesity; 3, male gender; 4, ambient humidity >60%; 5, apparent temperature >30°C; 6, ASA ≥ III; 7, diabetes mellitus; 8, history of post-traumatic arthritis; 9, discharge to rehabilitation/nursing facility; 10, advanced age; 11, history of patellar resurfacing; 12, lower income; 13, high postoperative drainage volume; 14, longer hospital stay; 15, previous surgical history of the affected site; 16, use of immunosuppressants; 17, hypoalbuminemia; 18, superficial infection; 19, bilateral TKA; 20, concomitant inflammatory disease (RA); 21, high drainage volume on postoperative day 1; 22, bacteriuria; 23, history of intra-articular injection; 24, anemia; 25, prolonged drainage tube retention; 26, chronic pulmonary disease; 27, intraoperative allogeneic blood transfusion; 28, antibiotic use >24 h; 29, Crohn's disease; 30, ulcerative colitis; 31, smoking; 32, alcohol abuse; 33, retained previous hardware; 34, CCI; 35, AVN; 36, lymphocytopenia; 37, low total cholesterol. ASA, American Society of Anesthesiologists Physical Status Classification System; CCI, Charlson comorbidity index; AVN, avascular necrosis of the femoral head; CC, case–control.

All the included studies describe the occurrence of PJI following primary TKA. Among them, five studies specifically explored the risk factors for PJI after both primary TKA and THA (20, 25, 28, 29, 33).

### 3.3 Quality of the studies included

The eligible articles were evaluated using the NOS, with scores used in place of the traditional star ratings for this analysis. One study scored 6 points (16), twelve studies scored 7 points (18–20, 22, 26–32, 34), five studies scored 8 points (15, 21, 23, 24, 33), and two studies scored 9 points (17, 25). The quality assessment of the included studies is presented in Table 2.

**Table 2:** Quality of the studies included.

Study inclusion	Case selection	Comparability	Outcome	NOS
Anis et al. (15)	4	1	3	8
Armit et al. (16)	4	0	2	6
Dowsey and Choong (17)	4	2	3	9
Guo et al. (18)	4	1	2	7
Iqbal et al. (19)	4	1	2	7
Jämsen et al. (20)	4	1	2	7
Claus et al. (21)	4	1	3	8
Siu et al. (22)	4	1	2	7
Remily et al. (23)	4	1	3	8
Suzuki et al. (24)	4	1	3	8
Wang et al. (25)	4	2	3	9
Cheng et al. (26)	4	1	2	7
Ning et al. (27)	4	1	2	7
Baoyu (28)	4	1	2	7
Di (29)	4	1	2	7
Tao et al. (30)	4	1	2	7
Zhe et al. (31)	4	1	2	7
Shaoqiang et al. (32)	4	2	1	7
Zhongren (33)	4	2	2	8
Du (34)	4	1	2	7

Study quality was assessed using the NOS for cohort studies. The NOS awards a maximum of nine stars across three domains: Selection of the study groups (0–4 stars), comparability of the groups (0–2 stars), and assessment of the outcome (0–3 stars). A higher total score (out of nine) indicates a higher methodological quality of the study.

### 3.4 Description of the risk factors

#### 3.4.1 The incidence of PJI after primary TKA

**Figure 2:**
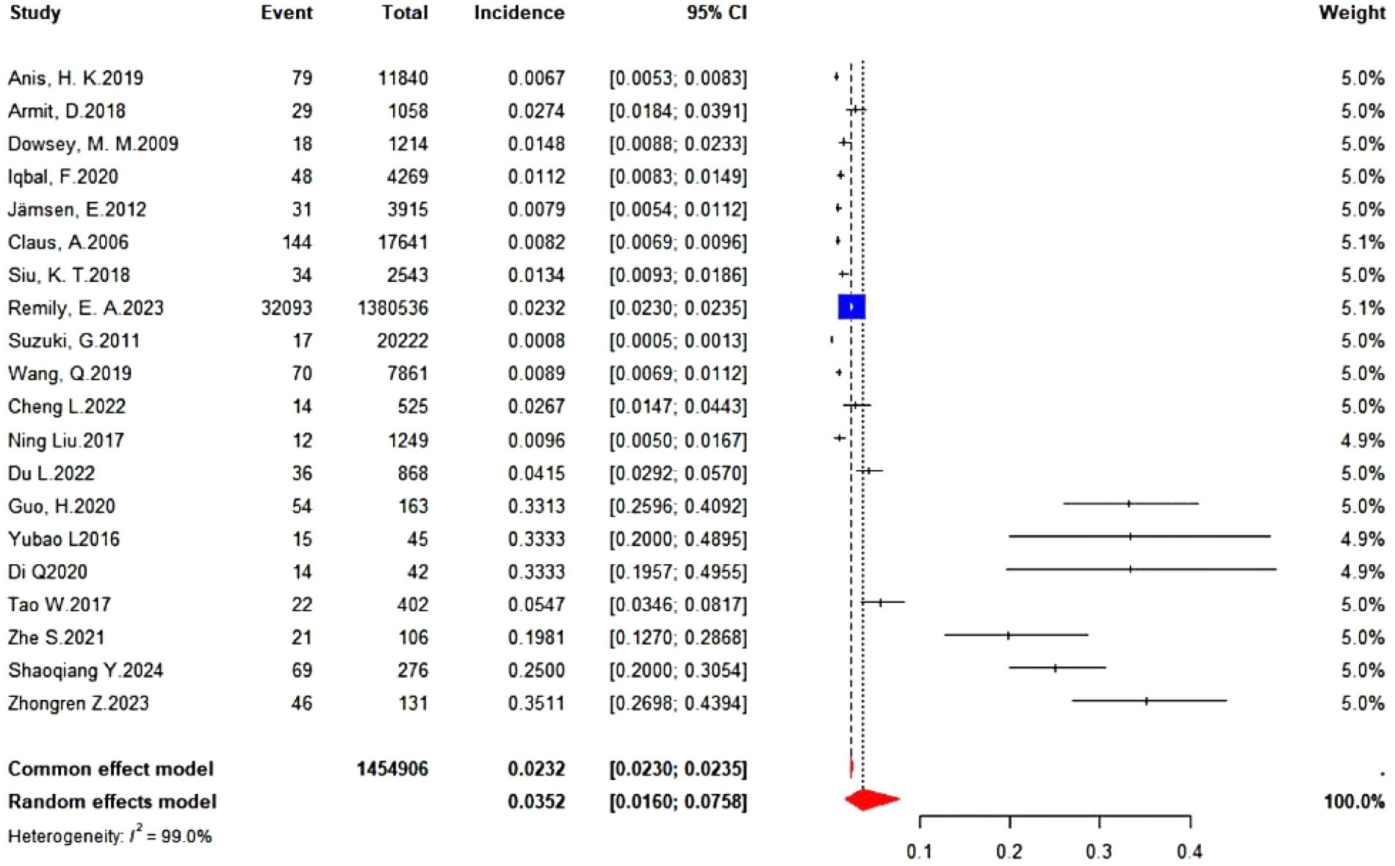
Analysis results of incidence rates from 20 studies.

The incidence rates from the 20 studies included in the synthesis via R4.5.1 were analyzed using a random-effects model to calculate the corresponding 95% confidence intervals (95% CI), weight values, and *I*^2^ for each study. The results indicated substantial heterogeneity among the studies (*I*^2^ = 99.0%), suggesting that the pooled incidence does not accurately represent the true rate of PJI following primary TKA. This discrepancy may be related to factors such as surgical duration, approach, technique, and patient status, among other factors. Additionally, seven of the studies were case–control studies (18, 28–33), which may not adequately reflect the incidence of PJI. The forest plot is shown in Figure 2.

#### 3.4.2 Longer operative time

**Figure 3:**
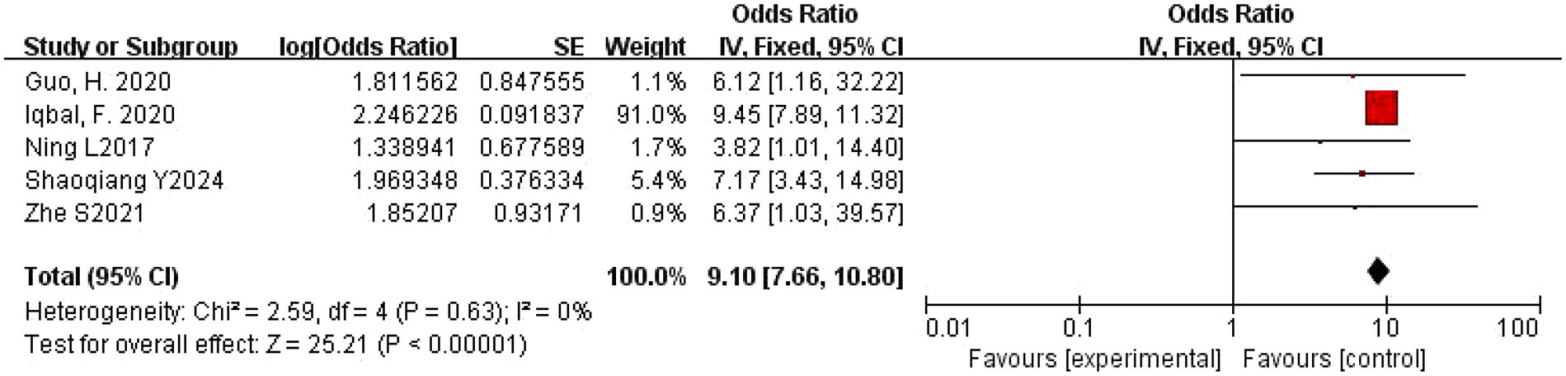
Forest plot of longer operative time and PJI following primary TKA.

A total of five studies were included (18, 19, 27, 31, 32). The pooled OR calculated using the fixed-effects model was 9.10 (95% CI: 7.66–10.80), with *I*^2^ = 0 and *P* = 0.63, indicating no heterogeneity among the studies. Therefore, it demonstrates that longer operative time is a risk factor for PJI following primary TKA. The forest plot is shown in Figure 3.

#### 3.4.3 Obesity

**Figure 4:**
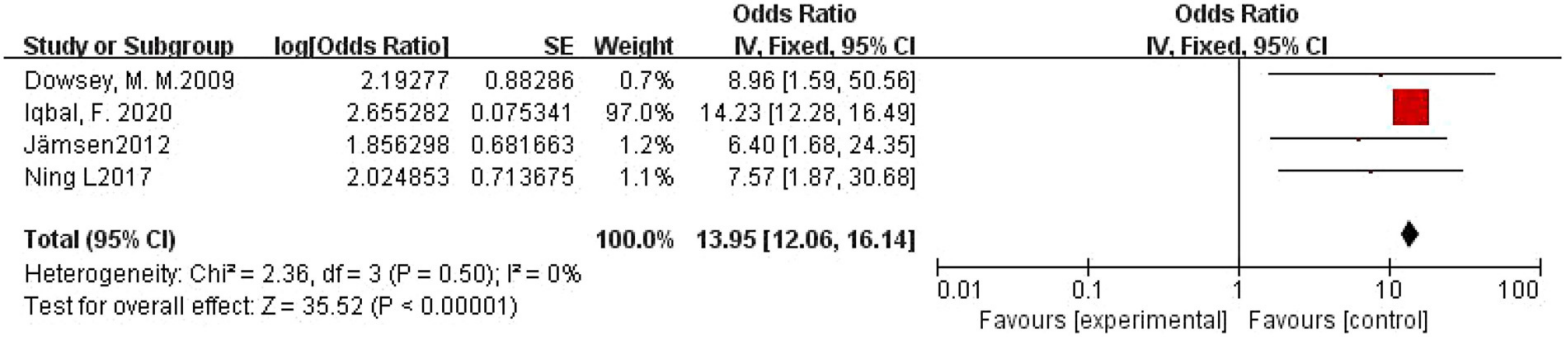
Forest plot of obesity and PJI following primary TKA.

A total of four studies were included (17, 19, 20, 27). The pooled OR calculated using the fixed-effects model was 13.95 (95% CI: 12.06–16.14), with *I*^2^ = 0 and *P* = 0.50, indicating no heterogeneity among the studies. Therefore, it demonstrates that obesity is a risk factor for PJI following primary TKA. The forest plot is shown in Figure 4.

#### 3.4.4 Male gender

**Figure 5:**
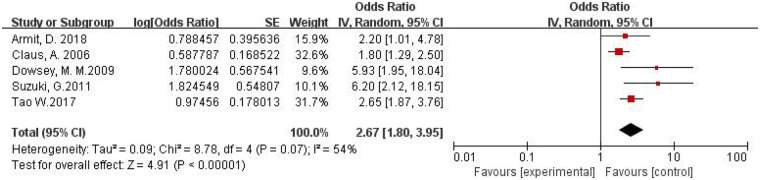
Forest plot of male gender and PJI following primary TKA.

A total of five studies were included (16, 17, 21, 24, 30). The pooled OR calculated by the random-effects model was 2.67 (95% CI: 1.80–3.95). The *I*^2^ value was 54% with a *P*-value of 0.07, indicating that the heterogeneity among the studies was within an acceptable range. This demonstrates that male gender is a risk factor for PJI following primary TKA. The forest plot for the meta-analysis of the association between male gender and PJI risk following primary TKA is shown in Figure 5.

#### 3.4.5 Diabetes

**Figure 6:**
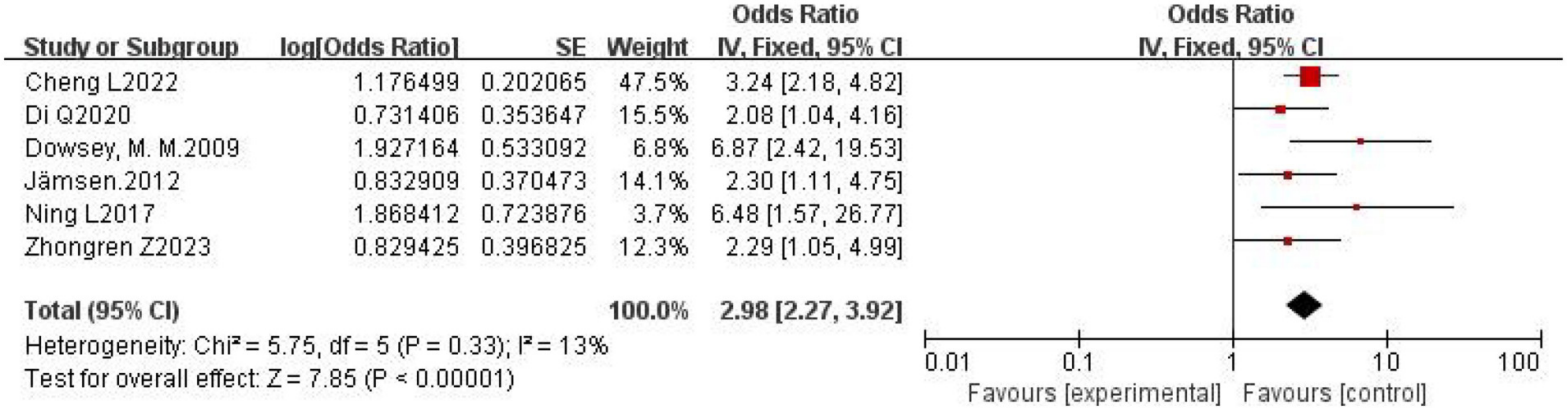
Forest plot of diabetes and PJI following primary TKA.

A total of six studies were included (17, 20, 26, 27, 29, 33). The pooled OR calculated using the fixed-effects model was 2.98 (95% CI: 2.27–3.92), with *I*^2^ = 13% and *P* = 0.33. The results indicate low heterogeneity among the studies, which is within an acceptable range. This demonstrates that diabetes is a risk factor for PJI following primary TKA. The forest plot is shown in Figure 6.

#### 3.4.6 Longer hospital stay

**Figure 7:**
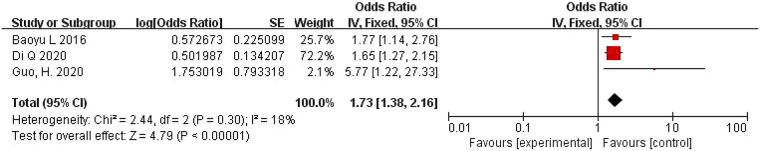
Forest plot of longer hospital stay and PJI following primary TKA.

A total of three studies were included (18, 28, 29). The pooled OR calculated using the fixed-effects model was 1.73 (95% CI: 1.38–2.16), with *I*^2^ = 18% and *P* = 0.30, indicating low heterogeneity among the studies, which is within an acceptable range. This demonstrates that a longer hospital stay is a risk factor for PJI following primary TKA. The forest plot is shown in Figure 7.

#### 3.4.7 Use of immunosuppressants

**Figure 8:**
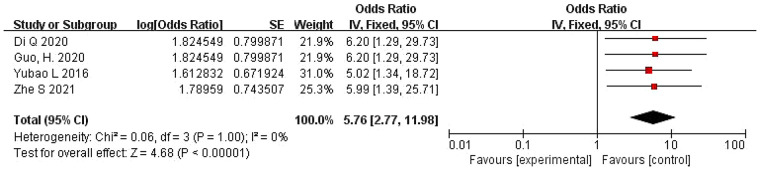
Forest plot of use of immunosuppressants and PJI following primary TKA.

A total of four studies were included (18, 28, 29, 31). The pooled OR calculated using the fixed-effects model was 5.76 (95% CI: 2.77–11.98), with *I*^2^ = 0 and *P* = 1.00, indicating no heterogeneity among the studies. Therefore, it demonstrates that the use of immunosuppressants is a risk factor for PJI following primary TKA. The forest plot is shown in Figure 8.

#### 3.4.8 Hypoalbuminemia

**Figure 9:**
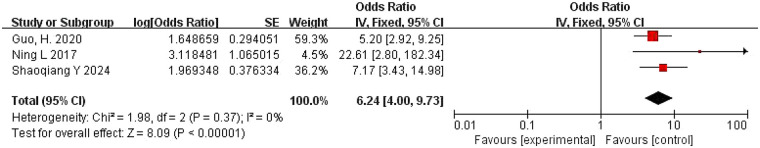
Forest plot of hypoalbuminemia and PJI following primary TKA.

A total of three studies were included (18, 27, 32). Pooled analysis using a fixed-effects model yielded an OR of 6.24 (95% CI: 4.00–9.73), with *I*^2^ = 0 and *P* = 0.37, indicating no heterogeneity among studies and suggesting that only sampling error was present. This confirms that hypoalbuminemia is a risk factor for PJI following primary TKA. The forest plot is presented in Figure 9.

#### 3.4.9 Underlying systemic inflammatory disease

**Figure 10:**
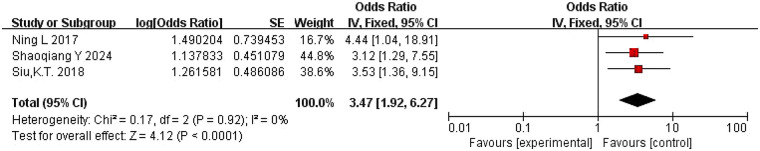
Forest plot of underlying systemic inflammatory disease and PJI following primary TKA.

A total of three studies were included (22, 27, 32). The pooled OR calculated using the fixed-effects model was 3.47 (95% CI: 1.92–6.27), with *I*^2^ = 0 and *P* = 0.92, indicating no heterogeneity among the studies. Therefore, it demonstrates that underlying systemic inflammatory disease is a risk factor for PJI following primary TKA. The forest plot is shown in Figure 10.

### 3.5 Pooled results from the meta-analysis of risk factors

Meta-analysis pooling all risk factors revealed that each factor had a *P*-value of <0.05, indicating statistically significant differences. This demonstrates that all nine factors are risk factors for PJI following primary TKA, as shown in Table 3.

**Table 3:** Results of the meta-analysis on risk factors associated with PJI following primary TKA.

Risk factor	Effect model	OR	95% CI	*I*^2^ (%)	*Z*	*P*
Longer operative time (Figure 3)	Fixed	9.10	7.66–10.80	0	25.21	<0.00001
Obesity (Figure 4)	Fixed	13.95	12.06–16.14	0	35.52	<0.00001
Male gender (Figure 5)	Random	2.67	1.80–3.95	54	4.91	<0.00001
Diabetes (Figure 6)	Fixed	2.98	2.27–3.92	13	7.85	<0.00001
Longer hospital stay (Figure 7)	Fixed	1.73	1.38–2.16	18	4.79	<0.00001
Use of immunosuppressants	Fixed	5.76	2.77–11.98	0	4.68	<0.00001
Hypoalbuminemia (Figure 8)	Fixed	6.24	4.00–9.73	0	8.09	<0.00001
Underlying systemic inflammatory disease (Figure 9)	Fixed	3.47	1.92–6.27	0	4.12	<0.0001

This table presents the pooled associations between potential risk factors and PJI, expressed as odds ratios (ORs) with 95% confidence intervals (95% CIs). OR > 1 indicates higher risk. A fixed-effects model was used when heterogeneity was low (*I*^2^ ≤ 50%); otherwise, a random-effects model was applied. The *I*^2^ statistic quantifies the percentage of total variation across studies due to heterogeneity. The *Z*-test and corresponding *P*-value assess the statistical significance of the pooled OR.

### 3.6 Heterogeneity analysis

Heterogeneity was observed among studies in all three analyses (Figures 4–6), but it remained within acceptable limits. All meta-analytic results were generated only after studies with substantial heterogeneity were excluded through sensitivity analysis. For example, in Figure 2, the final pooled outcome was obtained after excluding four studies (15, 25, 28, 29). The final pooled analysis of the five studies (18, 19, 27, 31, 32) showed no heterogeneity.

This review has certain limitations, which are reflected in the meta-analyses, particularly for the risk factors of longer operative time, obesity, and hypoalbuminemia (Figures 2, 3, 8). In these three meta-analyses, four (15, 25, 28, 29), eight (15, 18, 23–25, 28, 29, 34), and three (28, 29, 34) studies, respectively, were excluded due to significant heterogeneity. This heterogeneity may be attributed to clinical diversity, sample size variations, and variability in outcomes. Furthermore, the risk factors longer hospital stay, hypoalbuminemia, and underlying systemic inflammatory disease each included only three studies, which may lead to incomplete data. Nevertheless, all meta-analyses showed acceptable levels of heterogeneity among the included studies, and the final results are considered reliable.

## 4 Discussion

The results of this meta-analysis show that obesity has a combined OR value of 13.95 (Figure 3), indicating that this factor, particularly pathological obesity, is the most significant risk factor for PJI following primary TKA. Patients with obesity, especially pathological obesity, often have multiple comorbidities (17, 20), such as diabetes and cardiovascular diseases, as well as unhealthy lifestyle habits (e.g., smoking and alcohol abuse), which collectively influence surgical outcomes. Multiple studies have confirmed that obesity is an independent risk factor for PJI after TKA (35–37). Some studies have also demonstrated that the combination of obesity and diabetes significantly increases the risk of PJI, particularly in patients with pathological obesity (BMI ≥ 40 kg/m^2^) and diabetes. These patients tend to experience more wound complications, poor soft tissue healing, and greater surgical difficulty, which can prolong operative time. The meta-analysis revealed that longer operative time has a combined OR value of 9.10 (Figure 6), making it the second strongest risk factor. Prolonged surgery increases soft tissue damage due to anatomical traction and extends the exposure of peri-wound tissues to the environment, thereby elevating the risk of bacterial colonization and infection. Therefore, clinicians should consider whether such patients are suitable candidates for TKA or whether alternative interventions, such as weight and glycemic control, should be implemented prior to surgery to reduce operative time, which is crucial for minimizing PJI risk.

This study confirmed that hypoalbuminemia (OR = 6.24) and the use of immunosuppressants (OR = 5.76) are important risk factors (Figures 8, 9). Hypoalbuminemia is a marker of malnutrition, which directly impairs tissue repair capabilities and immunoglobulin synthesis, weakening the patient's systemic immune defense (38, 39). Similarly, long-term use of immunosuppressants systemically suppresses the patient's immune response, making them more susceptible to opportunistic infections, especially those with underlying systemic inflammatory diseases. The analysis also identified systemic inflammatory diseases (OR = 3.47) as a risk factor for PJI (Figure 9). Patients with inherent immune system dysregulation who require long-term immunosuppressive therapy for their primary condition face a “double hit”: they experience both abnormal immune inflammation and iatrogenic immunosuppression, significantly compromising their ability to combat pathogens introduced during surgery (40, 41). This underscores the importance of preoperative multidisciplinary assessment, optimized medication management, and enhanced nutritional support for these patients.

Furthermore, diabetes (OR = 2.98), male gender (OR = 2.67), and prolonged hospital stay (OR = 1.76) were also identified as significant risk factors (Figures 4–6). However, it is noteworthy that no PJI cases were reported in diabetic patients with a BMI < 30 kg/m^2^, whereas PJI cases were reported in diabetic patients with a BMI > 30 kg/m^2^, particularly those with pathological obesity (20). This suggests that in diabetic patients undergoing TKA, the effects of obesity and diabetes synergize, a finding supported by multiple studies (8, 9, 17, 20, 35). The exact mechanism behind male gender as a risk factor for PJI remains unclear but may be related to hormonal levels, social behavioral factors, or differences in comorbidities. Although a longer hospital stay may increase the risk of colonization with drug-resistant bacteria and cross-infection, especially in suboptimal ward conditions, it is more likely a consequence rather than a cause of PJI.

The strength of this study lies in the use of meta-analysis, which integrates data from multiple studies to provide higher-level evidence-based conclusions. All included factors exhibited highly significant *P*-values (*P* < 0.00001), indicating strong statistical validity (Figure 10). Additionally, the low heterogeneity (*I*^2^) for most factors (e.g., obesity, operative time, hypoalbuminemia, immunosuppressant use, and systemic inflammatory diseases) suggests consistent results across studies, enhancing the reliability of the conclusions. However, this study has certain limitations. First, the nature of meta-analysis means that the conclusions are constrained by the quality and methodological heterogeneity of the included studies. Second, most risk factors were analyzed as binary variables, preventing dose-response analysis (e.g., levels of glycemic control). Future prospective studies and in-depth investigations targeting specific high-risk populations are needed to develop more precise risk stratification models and personalized intervention strategies.

In summary, this meta-analysis demonstrates that PJI results from the combined effects of multiple factors. The most significant modifiable risk factors include obesity, operative time, nutritional status, immune status, and comorbid systemic inflammatory diseases. Surgeons should use this evidence to conduct comprehensive preoperative assessments and multidisciplinary optimization of patients, adhere to meticulous surgical techniques and aseptic principles during operations, and enhance postoperative monitoring. This multilayered defense strategy will ultimately reduce the incidence of PJI following primary TKA.

## 5 Conclusion

Based on Level II evidence from this systematic review and meta-analysis, surgeons should assess the identified risk factors present in patients. Periprosthetic joint infection is the most serious complication of total joint arthroplasty in clinical practice, and inadequate management can impose a significant burden on both patients and healthcare institutions. Therefore, the evidence synthesized here supports the development of personalized, preoperative optimization plans aimed at mitigating these risks to improve surgical success and prevent PJI.

## Data Availability

The raw data supporting the conclusions of this article will be made available by the authors, without undue reservation.
